# Supplementary figures and images for: Divergent metabolism between Trypanosoma congolense and Trypanosoma brucei results in differential sensitivity to metabolic inhibition
Source: PLoS Pathog. 2021 Jul 26;17(7):e1009734. doi: 10.1371/journal.ppat.1009734 (PMC8384185; doi:10.1371/journal.ppat.1009734)

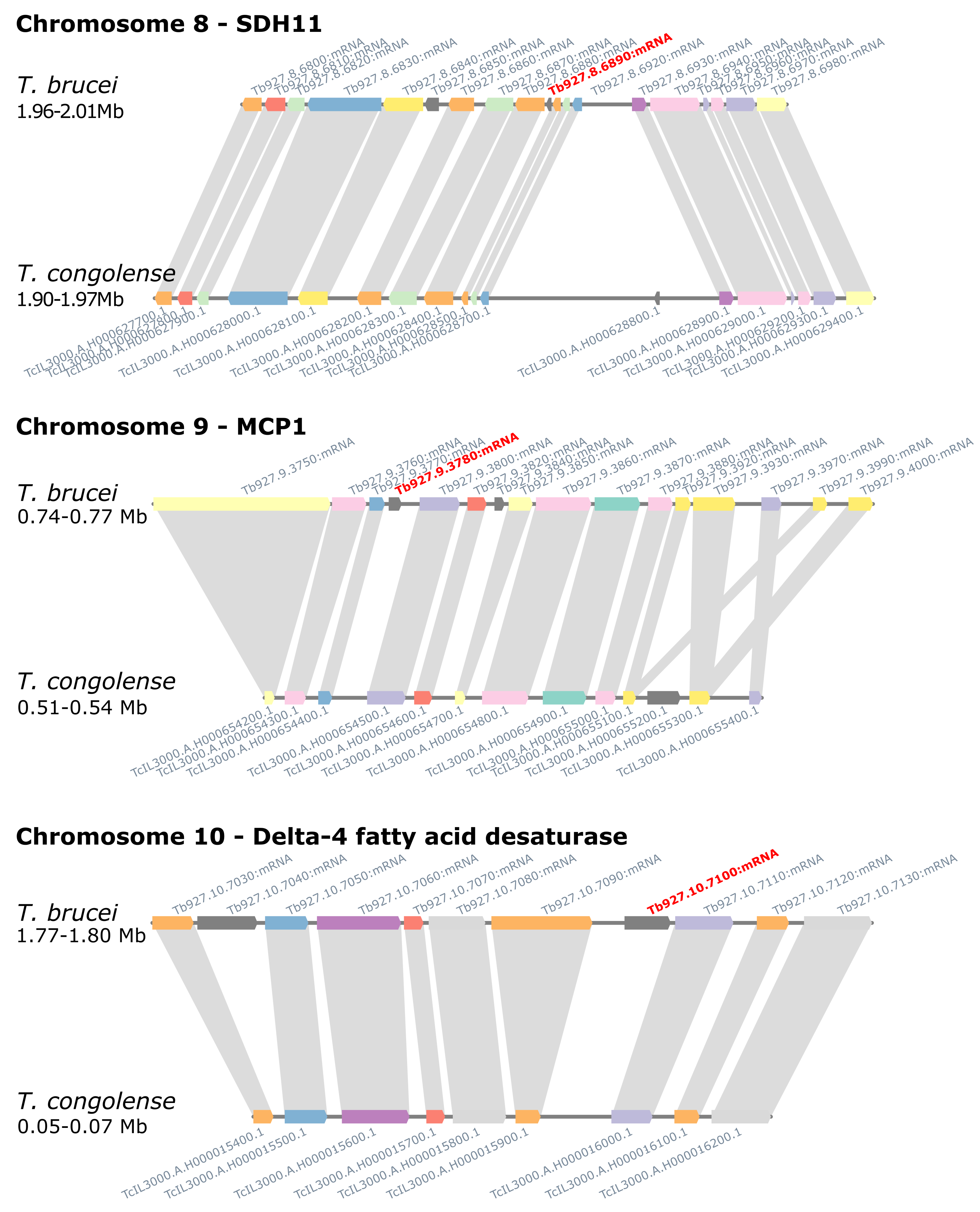

Supplement: S1 Fig — Plots were generated to assess levels of synteny between the species in regions where genes appear to be absent in T. congolense. For 3 genes, SDH11 (top), MPC1 (middle) and a putative delta-4 fatty acid desaturase (bottom), surrounding regions are highly syntenic between T. brucei and T. congolense, indicating that these are likely 3 deletions from the T. congolense genome. Whilst this does not rule out existence of these genes in other genomic regions, approaches such as orthoMCL and BLAST did not yield high probability orthologues in T. congolense. (TIFF) [file ppat.1009734.s001.tiff]

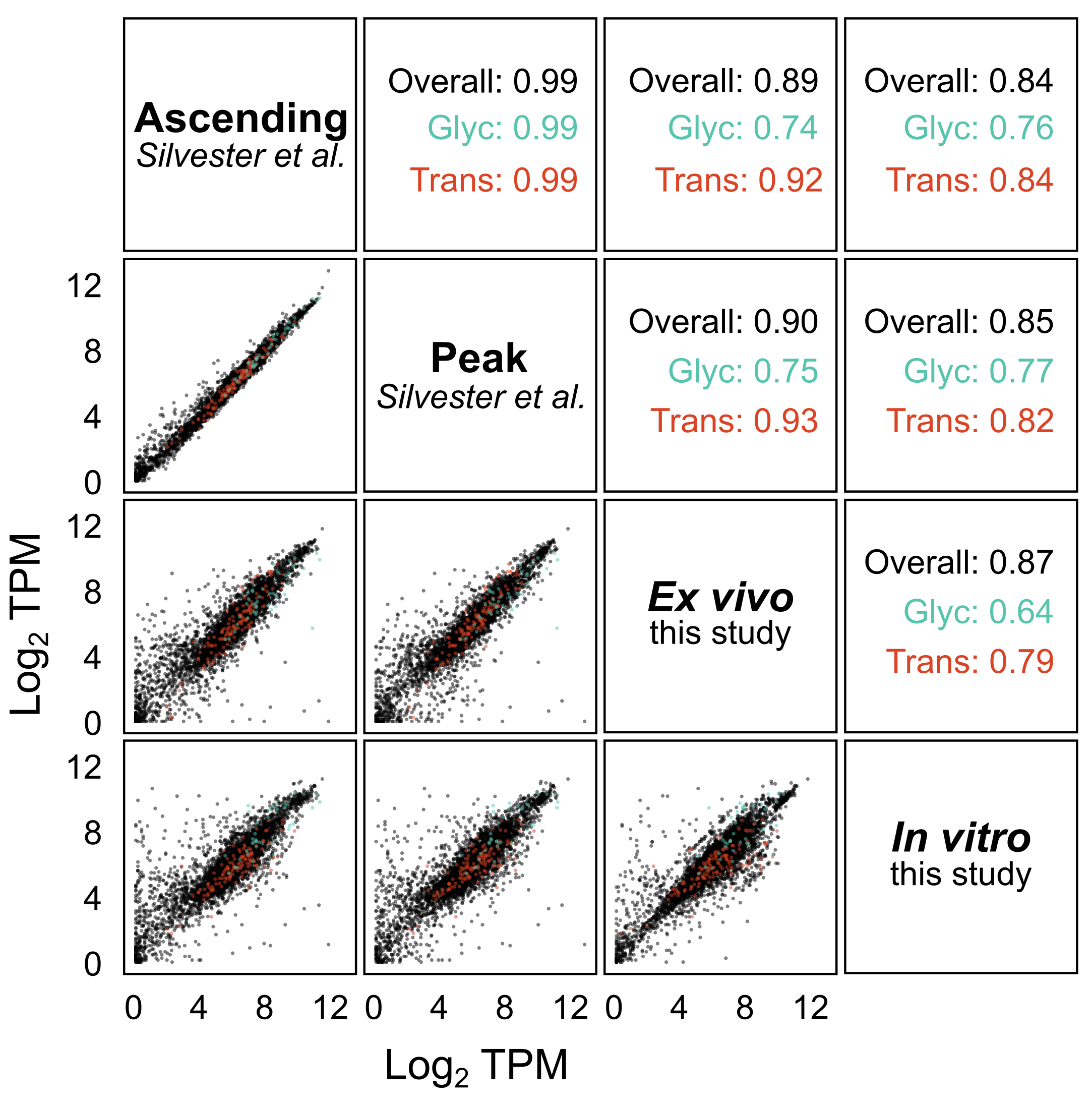

Supplement: S2 Fig — Scatter matrix of T. congolense datasets from this study compared to ascending and peak parasitaemia in vivo transcriptomics data generated by Silvester and colleagues [59]. TPM values were calculated for each gene in the T. congolense genome (S1 Table) and log2(TPM+1) was plotted. Lower panels: Scatter plots of individual comparisons of the 4 datasets. Red dots correspond to genes associated with glycolysis, green dots correspond to genes possessing transmembrane domains that are likely to be transporters; Diagonal panels: sample names; Upper panels: Pearson correlation coefficients for comparisons of entire datasets (black), glycolytic pathway (“Glyc”, green) and proteins with predicted transmembrane domains (“Trans”, red). (TIFF) [file ppat.1009734.s002.tiff]

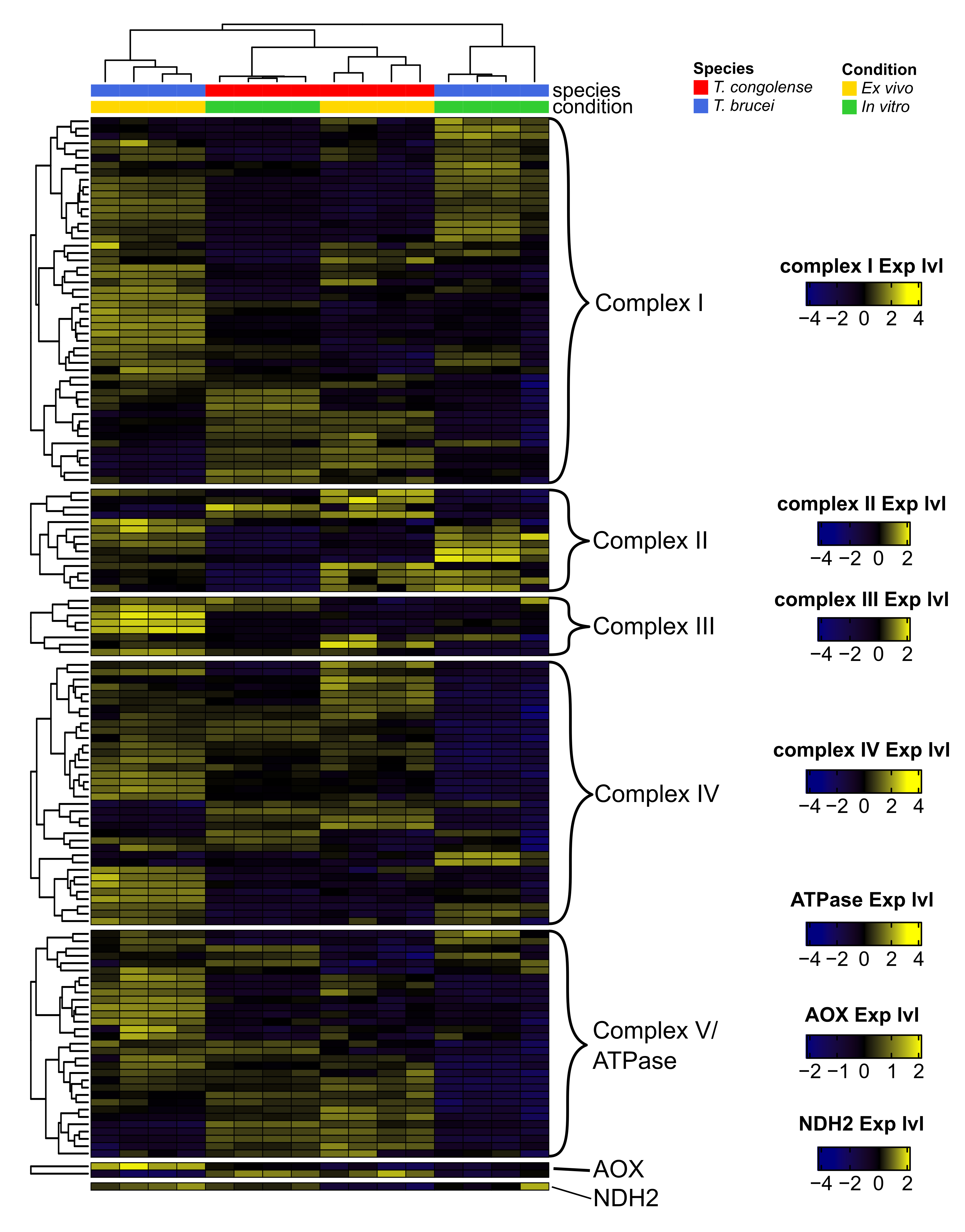

Supplement: S3 Fig — A heatmap of all ETC complexes based on a table generated by Zikova and colleagues [76]. Heatmaps are divided into the alternative oxidases (AOX), NADH dehydrogenase 2 (NDH2), complex I, II, III, IV and ATPase (complex V). (TIFF) [file ppat.1009734.s003.tiff]

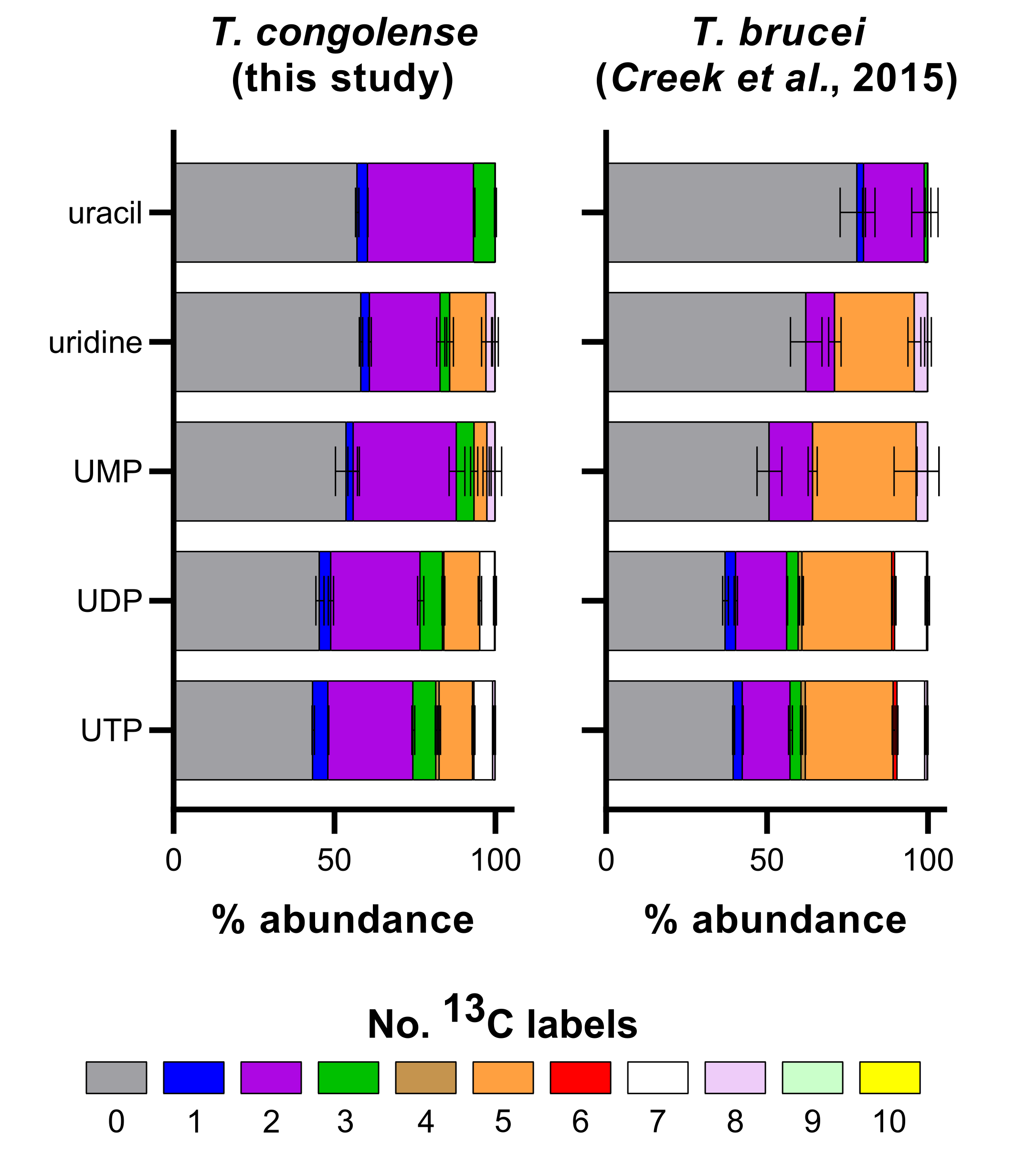

Supplement: S4 Fig — Comparative analysis of glucose-derived pyrimidine labelling in T. congolense and T. brucei (taken from [45]). (TIFF) [file ppat.1009734.s004.tiff]

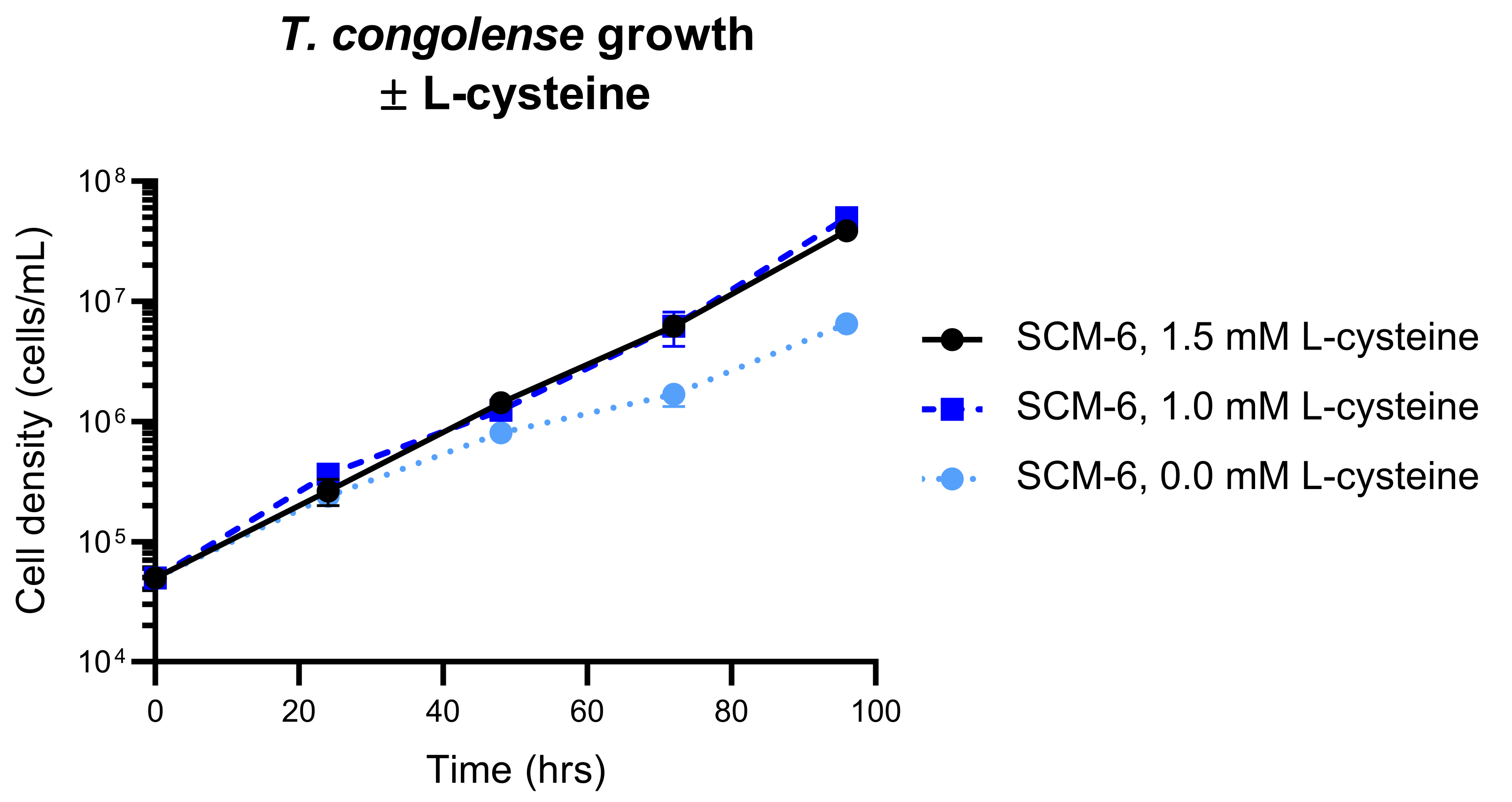

Supplement: S5 Fig — Parasites were grown in SCM-6 supplemented with 1.5 mM, 1.0 mM or absence of L-cysteine. Cell density was monitored every 24 hours. (TIFF) [file ppat.1009734.s005.tiff]

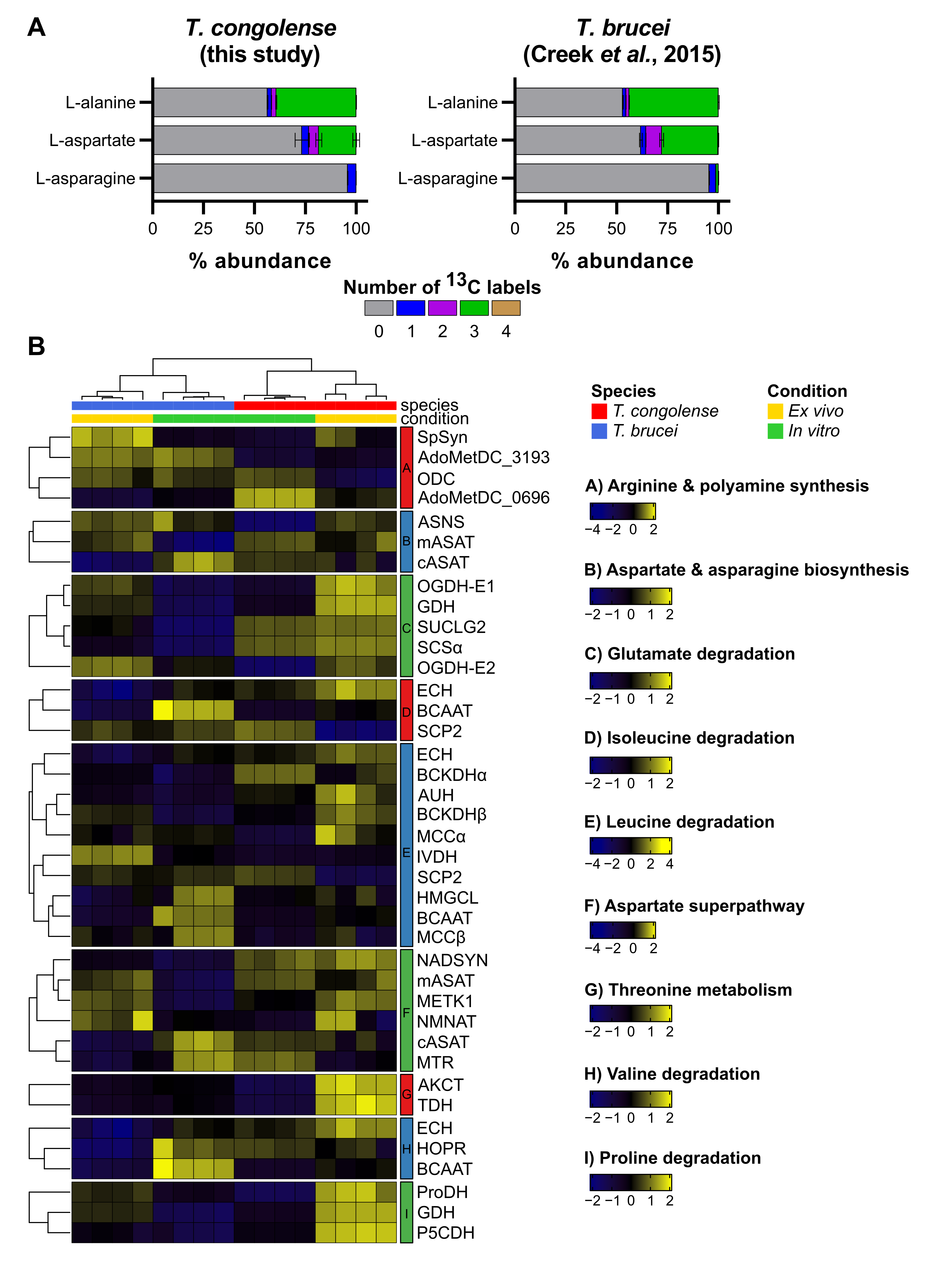

Supplement: S6 Fig — A) glucose-derived carbon labelling of amino acids B) Transcriptomics pathway analysis. TrypanoCyc pathways and gene IDs: A) Arginine and polyamine synthesis (ARG+POLYAMINE-SYN): SpSyn, spermidine synthase, TbTc_1034; AdoMetDC_3193, AdoMet decarboxylase, TbTc_3193; ODC, ornithine decarboxylase, TbTc_5903; AdoMetDC_0696, AdoMet decarboxylase, TbTc_0696. B) Aspartate and asparagine biosynthesis (ASPASN-PWY): ASNS, asparagine synthetase, TbTc_4894; mASAT, mitochondrial aspartate aminotransferase, TbTc_5877; cASAT, cytosolic aspartate aminotransferase, TbTc_0799. C) Glutamate degradation (GLUCAT-PWY): OGDH-E1, 2-oxoglutarate dehydrogenase E1, TbTc_2864; GDH, glutamate dehydrogenase, TbTc_0872; SUCLG2, succinyl-CoA ligase, TbTc_3392; SCSα, succinyl-CoA synthetase, TbTc_0813; OGDH-E2, 2-oxoglutarate dehydrogenase E2, TbTc_3057. D) Isoleucine degradation (ILEUDEG-PWY): ECH, enoyl-CoA hydratase, TbTc_3283; BCAAT, branched-chain amino acid aminotransferase, TbTc_0559; SCP2, 3-ketoacyl-CoA thiolase, TbTc_4024. E) Leucine degradation (LEUDEG-PWY): ECH, enoyl-CoA hydratase, TbTc_3283; BCKDHα, 2-oxoisovalerate dehydrogenase α, TbTc_1182; AUH, methylglutaconyl-CoA hydratase, TbTc_5348; BCKDHβ, 2-oxoisovalerate dehydrogenase β, TbTc_0682; MCCα, 3-methylcrotonyl-CoA carboxylase α, TbTc_1670; IVDH, isovaleryl-CoA dehydrogenase, TbTc_3112; SCP2, 3-ketoacyl-CoA thiolase, TbTc_4024; HMGCL, hydroxymethylglutaryl-CoA lyase, TbTc_6160; BCAAT, branched-chain amino acid aminotransferase, TbTc_0559; MCCβ, 3-methylcrotonyl-CoA carboxylase β, TbTc_5385. F) Aspartate superpathway (PWY0-781): NADSYN, NAD+ synthase, TbTc_2404; mASAT, mitochondrial aspartate aminotransferase, TbTc_5877; METK1, AdoMet synthase, TbTc_0178; NMNAT, nicotinamide/nicotinic acid mononucleotide adenylyltransferase, TbTc_4133; cASAT, cytosolic aspartate aminotransferase, TbTc_0799; MTR, 5-methyltetrahydropteroyltriglutamate-homocysteine S-methyltransferase, TbTc_5805. G) Threonine degradation (PWY1V8-11): AKCT, 2-amino-3-k [file ppat.1009734.s006.tiff]

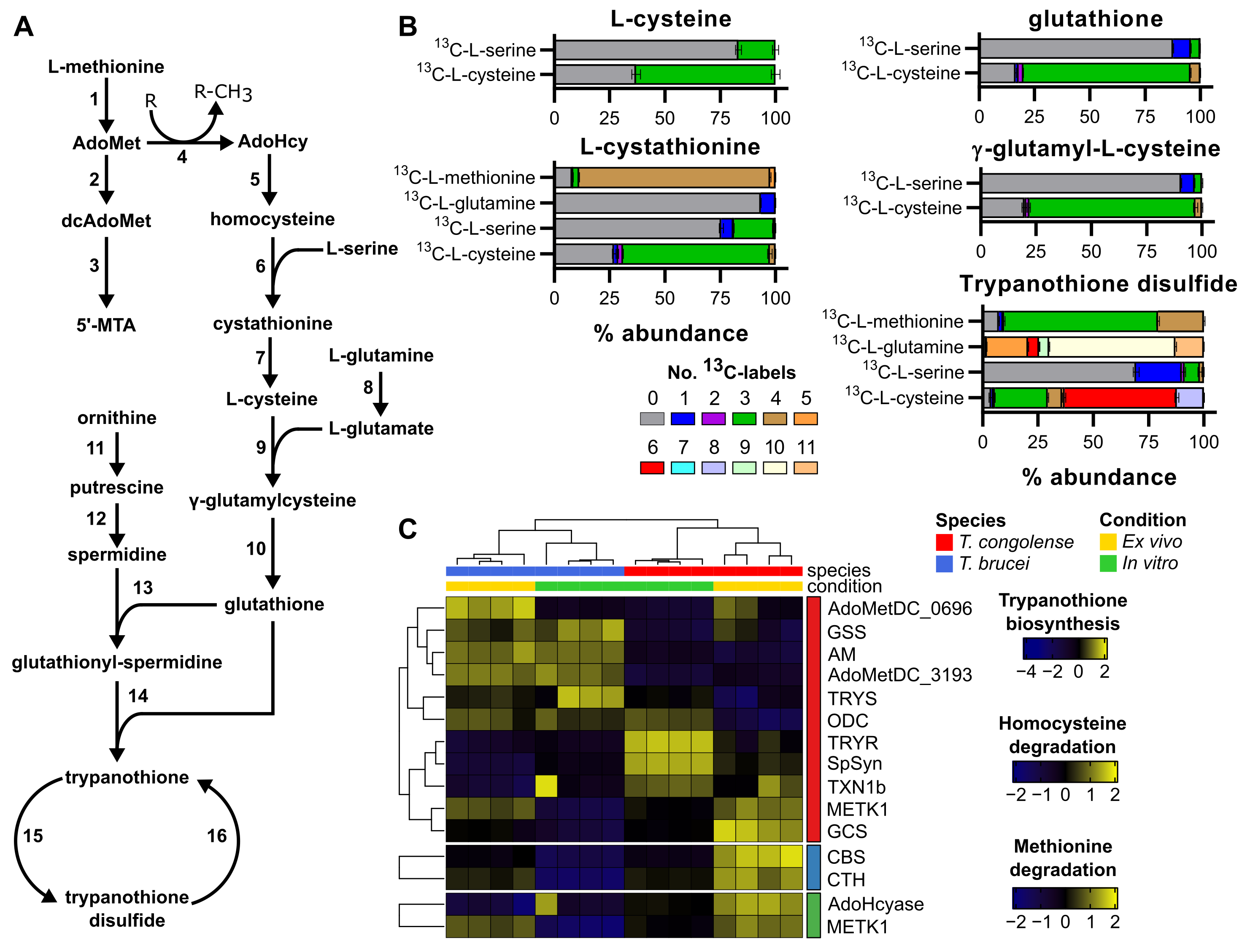

Supplement: S7 Fig — Metabolomics and transcriptomics analyses were carried out to analyse trypanothione biosynthesis. A) A simplified map of trypanothione biosynthesis as known in T. brucei. Numbers refer to the following enzymes: 1, S-adenosyl-L-methionine synthase, METK1; 2, S-adenosyl-L-methionine decarboxylase, AdoMetDC; 3, spermidine synthase, SpSyn; 4, methyltransferase reaction, MTase; 5, S-adenosyl-L-homocysteine dehydrolase, AdoHycase; 6, cystathionine beta synthase, CBS; 7, cystathione gamma lyase, CTH; 8, glutaminase/amidase, AM; 9, gamma-glutamylcysteine synthetase, GCS; 10, glutathione synthetase, GSS; 11, ornithine decarboxylase, ODC; 12, spermidine synthase, SpSyn; 13, glutathionylspermidine synthase, GSP; 14, trypanothione synthetase, TRYS; 15, tryparedoxin peroxidase, TXN1b; 16, trypanothione reductase, TRYR. B) Isotopologue labelling experiments using 100% 13C-L-serine, 13C-L-glutamine, 13C-L-methionine or 13C-L-cysteine, showing the abundance of carbon labelling derived from these amino acids in components of the trypanothione biosynthesis pathway. C) Transcriptomics analysis. TrypanoCyc pathways and gene IDs: Trypanothione biosynthesis (PWY1V8-6): AdoMetDC_0696, S-adenosylmethionine decarboxylase, TbTc_0696; GSS, glutathione synthetase, TbTc_3678; AM, amidase, TbTc_5549; AdoMetDC_3193, S-adenosylmethionine decarboxylase, TbTc_3193; TRYS, trypanothione synthetase, TbTc_1359; ODC, ornithine decarboxylase, TbTc_5903; TRYR, trypanothione reductase, TbTc_4239; SpSyn, Spermidine synthase, TbTc_1034; TNX1b, tryparedoxin 1b, TbTc_0324; METK1, S-adenosylmethionine synthetase, TbTc_0178; GCS, gamma-glutamylcysteine synthetase, TbTc_3424. Homocysteine degradation/cysteine biosynthesis (HOMOCYSDESGR-PWY1): CBS, cystathionine beta synthase, TbTc_0413; CTH, cystathione gamma lyase, TbTc_1051. Methionine degradation I (METHIONINE-DEG1-PWY): AdoHcyase, S-adenosylhomocysteine hydrolase, TbTc_0685; METK1, S-adenosylmethionine synthase, TbTc_0178. (TIFF) [file ppat.1009734.s007.tiff]

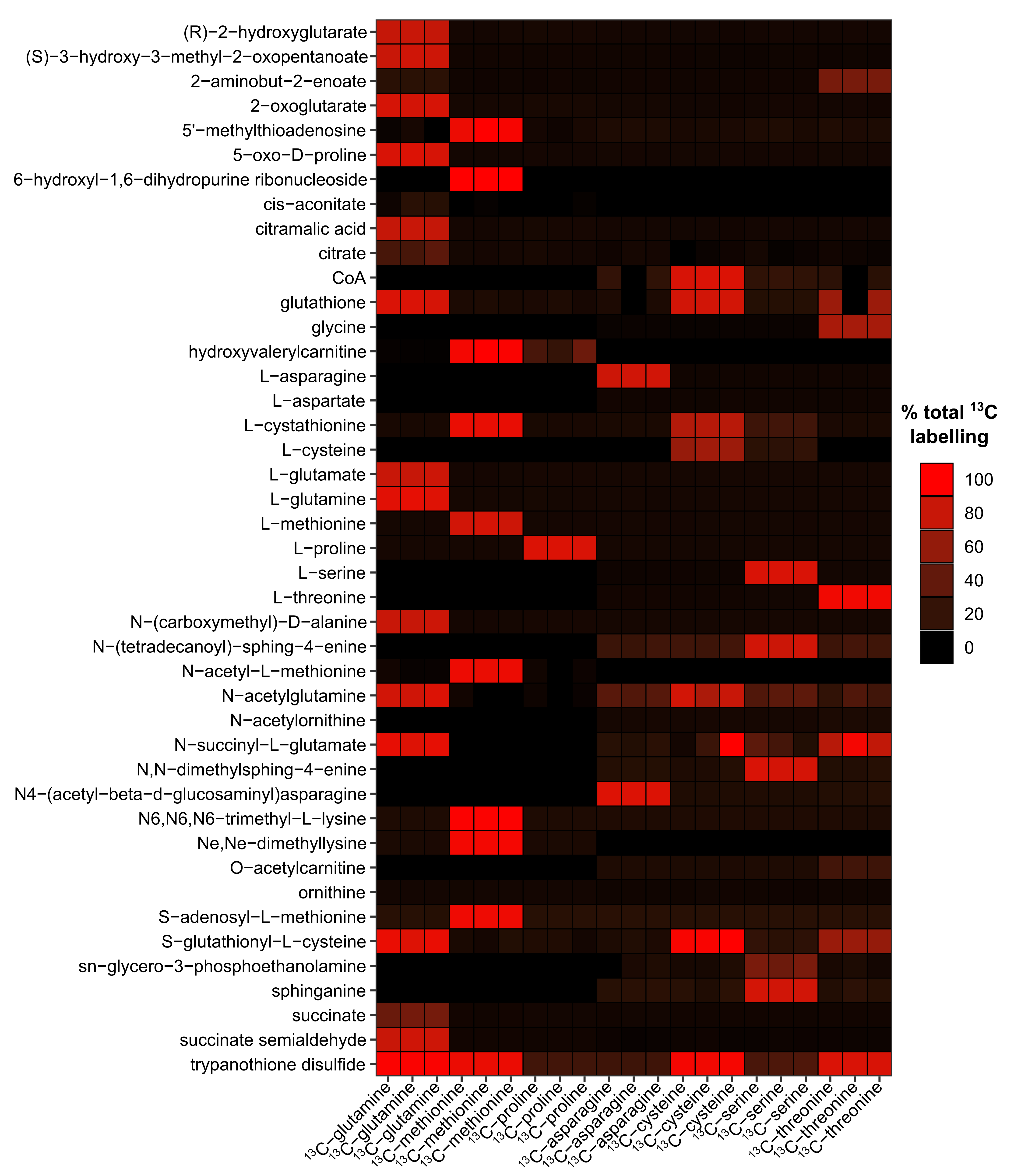

Supplement: S8 Fig — Percentage total labelling of metabolites identified in data from 6 stable isotope labelling experiments using 13C-L-asparagine, 13C-L-cysteine, 13C-L-glutamine, 13C-L-methionine, 13C-L-proline and 13C-L-serine. Colour intensity correlates to the total fraction of the metabolite that was 13C-labeled. (TIFF) [file ppat.1009734.s008.tiff]

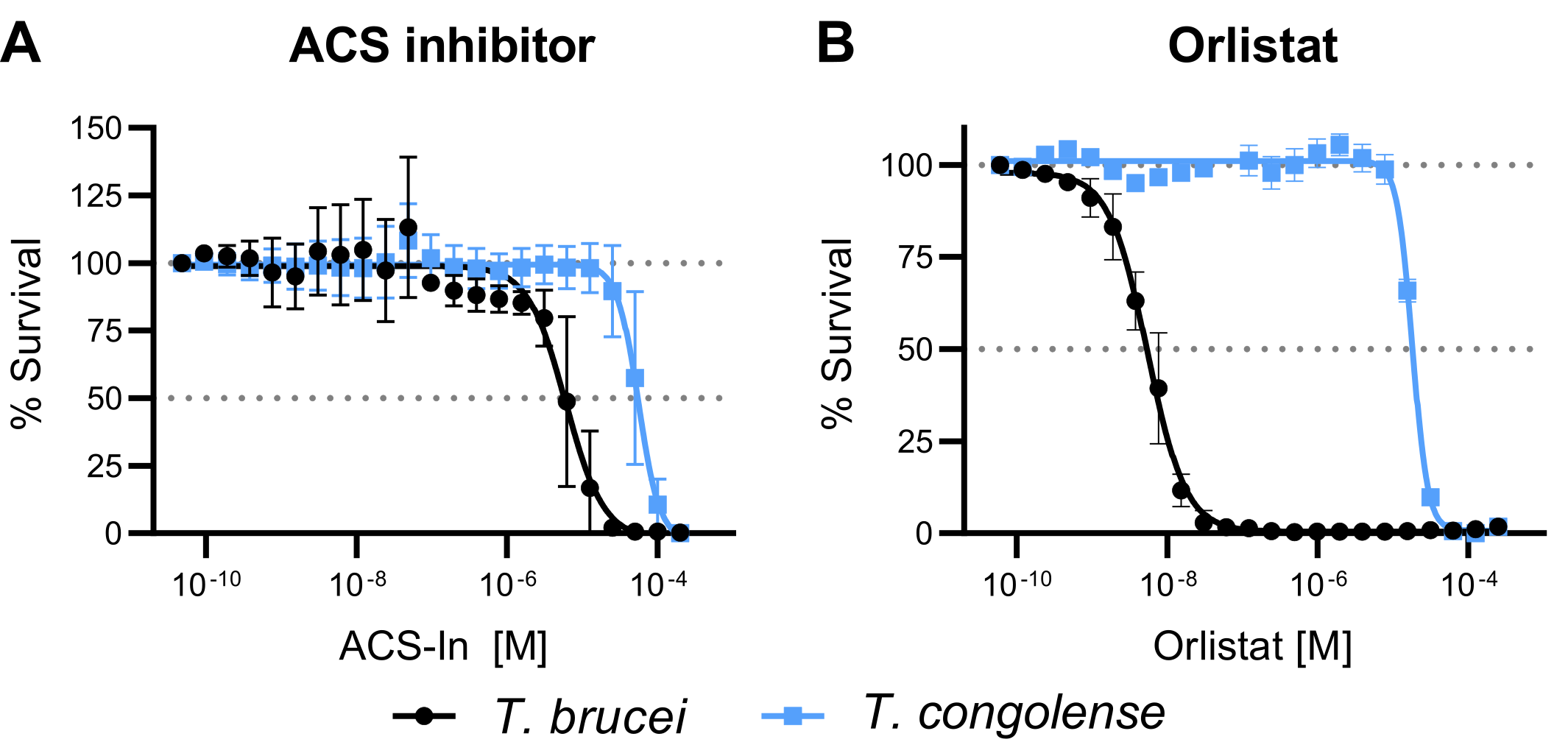

Supplement: S9 Fig — Sigmoidal dose-response curves to determine differential sensitivity of the two species of parasite to inhibition of an ACS inhibitor (panel A) and Orlistat (B). (TIFF) [file ppat.1009734.s009.tiff]
