# Supplementary material for: Divergent metabolism between Trypanosoma congolense and Trypanosoma brucei results in differential sensitivity to metabolic inhibition
Source: PLoS Pathog. 2021 Jul 26;17(7):e1009734. doi: 10.1371/journal.ppat.1009734 (PMC8384185; doi:10.1371/journal.ppat.1009734)
Supplement: Attachment — Submitted filename: Steketee_et-al_reviewer_rebuttals.pdf [file ppat.1009734.s016.pdf]

THE ROSLIN INSTITUTE
The University of Edinburgh
Easter Bush
Midlothian
EH25 9RG
Telephone: +44 (0)131 651 9100
www.ed.ac.uk/roslin

Dear Prof. Clayton and PLoS Pathogens editorial team,

Thank you for your reviews of our manuscript "Divergent metabolism between Trypanosoma congolense and Trypanosoma brucei results in differential sensitivity to metabolic inhibition". We are pleased with the response to our work.

Although both editor and reviewers seemed to find the work of interest, there were some major issues raised, in particular regarding cross-species transcriptomics analysis. These were very constructive comments, so thanks to all involved, and in the revised manuscript we present a complete reanalysis of these data with statistical treatment. Importantly, the major findings and conclusions of the manuscript are unchanged by this reanalysis, but we believe it provides an improved treatment of the data that could be used in future cross-species analyses. We have also addressed each individual point raised by the editor and all three reviewers. In addressing these points, we have revised and expanded the manuscript in particular by i) revising the transcriptomics analysis and statistics (including a new supplementary table S1, and modifying figures 1, 3, 5, 7, 8, S2 (previously S1), S3, S6 and S7), ii) insertion of a new supplementary figure examining syntenic genome regions (S1), iii) clarification of the text as suggested by the reviewer comments, especially with respect to metabolomics, iv) down-playing the "drug sensitivity" aspects of the manuscript, including the title and abstract, v) removing two RNAi experiments as requested by reviewer 1 and revising figure 4, and vi) addressing grammatical and punctuation errors.

Thank you for considering our manuscript, we hope the revised manuscript is suitable for publication in PLoS Pathogens.

Kind regards,

Pieter Steketee

Michael Barrett

Liam Morrison

Director of The Roslin Institute and Dean of Research Royal (Dick) School of Veterinary Studies Professor Eleanor Riley
The Roslin Institute is associated with the Royal (Dick) School of Veterinary Studies, the University of Edinburgh
The Roslin Institute receives strategic funding from the Biotechnology and Biological Sciences Research Council (BBSRC)
The University of Edinburgh is a charitable body, registered in Scotland, with registration number SC005336

Editor:
Line 181 - why the lower alignment % of T. brucei reads - was it because the genome used for the alignment didn't include relevant VSGs?

This study used the T. b. brucei cell line STIB 247 (PMID: 4144952, 4144955, 4144956). As suggested, the TREU 927 reference genome will lack the relevant VSG repertoire for this strain, and this is the most likely explanation for the lower % of T. brucei reads aligning to the TREU 927 reference genome. Unfortunately, to our knowledge there is no suitable STIB 247 annotated assembly that could be substituted here. However, it should be noted that outside of the VSG, the majority of reads from housekeeping genes should map without issue. We have inserted a sentence to clarify this on lines 188-189.

Line 183: "to minimize artefacts from multigene families, only uniquely aligned reads were used for downstream analyses". You can't do this since it excludes many very important genes, e.g. glycerol kinase, aldolase.... For PGKs you can use the 3'-UTRs as well. Please also analyse the multi-gene families - or at least, those that are important. For known important families you can simply sum the reads that come from the different paralogues. If you remove all multipy aligned reads you also get completely distorted TPM values.
Transcriptomes must be compared using an appropriate tool designed for such analyses such as EdgeR or DeSeq2.

We apologise. Text in lines 183-184 of original manuscript reflected an older analysis than that presented and did not accurately reflect the data shown (which did not exclude non-uniquely mapping reads). However, the point raised by the editor is a really important one, and in our revised manuscript we include a reanalysis of the sequencing data (detailed below). The reanalysed data closely match those shown previously (Pearson correlation coefficient <0.99), but deal with the multigene/cross-species comparison better.

Our revised analysis maps reads with HiSat2 --no-splice-alignment, as previously, and also -k 1 allowing each read to map only once (even if more than one map were possible). This distributes reads from near-identical regions of multi-copy genes between all copies (averaging out reads across the family but maintaining the total count). When summing across paralogues to allow comparison between species, the TPM value in each organism should therefore be an accurate representation of the total abundance of transcripts originating from a gene family, without artefacts from either counting multi-mapping reads several times or excluding them from count. In addition to revision of scatter plots and correlation coefficients for figures 1 and S2 (previously S1), figures 3, 5, 7, 8, S3, S6 and S7 include data from this revised analysis.

With regards to UTRs, unfortunately these are not annotated in the T. congolense genome and cannot be included for this organism. We feel that including UTRs in the read counts for T. brucei but not for T. congolense would add further inconsistency to a cross-species comparison that is already challenging. Therefore, we took the decision to exclude UTRs for T. brucei in order to keep the analysis as consistent as possible between the two species. We have added a sentence into the M&M section (lines 1171-1174) to explain these choices, which reads: "“CDS” was chosen as feature for read counting instead of “exon”, as UTRs are not annotated in the T. congolense genome compared to the T. brucei TREU 927 genome, which could lead to further discrepancies during cross-species transcriptome analysis.”

Regarding statistical treatment of the data: in order to make comparisons between species it is necessary to account for difference in transcript lengths (or gene model lengths) between the organisms/assemblies (using our orthoTPM approach). Unfortunately, edgeR and DESeq2 incorporate raw read count data into their models, so cannot be used to analyse transformed datasets. However, the editor’s point about analysis is well made. In the revised manuscript we have applied the models provided in the limma package (PMID: 25605792) to provide statistical support to the observed changes in the transcriptomes between the species. These methods are very similar to those of edgeR and DESeq2, but can be applied to non-raw count data. Gene/families with low orthoTPM counts (98 in total) were removed, and orthoTPM values were

log-transformed (log$_2$[TPM+1]) before analysis using the eBayes() function (trend = TRUE, robust = TRUE). These data are included in the revised manuscript and are provided in the new S1 table.

We have amended the methodology, results and discussion in the revised manuscript to reflect these analyses, which we believe are an improvement on the original. However, we would like to note that these changes do not substantially affect the findings of conclusions discussed.

What are the relative division times of T. congolense and T. brucei in culture? Could this account for some differences in e.g. glucose consumption? Also transcriptomes obtained at peak parasitaemia are not comparable to those obtained in growing cells. To compensate for this I suggest that instead of thhe T. brucei transcriptome that was taken when the cells were too dense, you use some of the many publicly available datasets for bloodstream-form T. brucei that were not so dense - ideally, less than 2 million per ml. Cell densities of the parasites used for RNASeq must be stated, and the stage in the growth curve given. (Ideally this should be early-to-mid log phase and at least 2 divisions before the maximum density.)

Apologies for these oversights. Division times of T. congolense and T. brucei Lister 427 used here are 11-12 h and 6-7 h, respectively (we have now included this information in lines 393-394 and 1077-1079). It is correct that this could impact consumption/excretion of metabolites, and for this reason, most analysis of supernatant metabolomics assaying was concentrated on T. congolense alone. However, establishing differences in glucose consumption between the species was crucial, and growth differences between the species were specifically taken into account in the model used (see PMID: 26491423) to calculate the rate of glucose uptake. Cell density was recorded at each time point where supernatant samples were taken, and these data were used in the model along with time and glucose concentration.

For RNAseq analysis we specifically chose the T. brucei STIB 247 strain in order to incorporate a pleomorphic cell line in our analyses. We have added text to the results (lines 180-182) and M&M (lines 964-967) sections to clarify cell density and growth stage, the latter of which reads: "For in vitro parasite RNA isolation, samples of both T. congolense (IL3000) and T. brucei (STIB 247) were taken from actively dividing cultures grown to densities of 1.8 – 2 × 10^6 cells/mL."

Line 306 You cannot trust the gene copy numbers in the T. brucei 927 assembly because, due to the way in which is was generated, identical repeats are eliminated. See BMC Genomics volume 16, Article number: 1118 (2015) (Table S1) for some numbers. For example, 927 has 4 copies of glycerol kinase, and other strains (including field isolates) up to 9.

This is a very good point. Upon further discussion, we have decided to remove the sentence discussing gene copy numbers from the revised manuscript. Please note that this has not substantially affected any of our analyses or conclusions.

Table S1 -
1. Please include a raw data table (mapped reads for all genes, not just the unique reads) as a separate sheet.
2. Please round RPMs appropriately (e.g. to 1 decimal place) - makes it much easier to see differences.

In our revised manuscript, we include further supplementary tables containing raw read counts (HTSeq-count output, RPK counts and TPM counts) generated prior to the Orthofinder step for cross-species analysis. These tables are now within the same excel file as the final cross-species dataset, in worksheets labelled "Raw_reads", "RPKs" and "TPMs".

We have rounded the TPM values to 1 decimal place for clarity, although in the main text we are still quoting log$_2$ fold changes to 2 decimal places.

1. Title and abstract should be re-worded -since it is misleading. Instead of "drug sensitivity" it should be "sensitivity to metabolic inhibitors" since most of the compounds used are not used as anti-trypanosomal drugs - or in some cases, not used as drugs at all. I agree with the reviewer who says that this part should be very much down-played. The most that you can say is that these results will help in selecting compounds that work on all species. The proportions of different species are quite variable across Africa; no African farmer is interested in having a drug that treats only T. congolense, so no company is going to try to market such a drug.

We have amended the title and abstract as suggested by the editor. While we very much agree with the points raised regarding selection of compounds that will work on all species as a preferred goal, it is worth noting that the recent drug development efforts (e.g. GALVmed & benzoxaboroles) have focused on T. congolense and T. vivax only, and therefore highlighting potential targets in T. congolense we believe is still useful, as these may prove to also be viable targets in T. vivax. However, we accept the editor's point and have amended accordingly.

Line 141 - typo - "BSF T. congolense primarily expresses cytosolic PGK-C, rather than glycosomal PGK-B". Wrong way round, PGKC is glycosomal.

This has been amended.

Part II – Major issues:

Reviewer #3:

The major issues described by reviewer 3 were primarily concerns associated with analysis of metabolomics data, and particularly identification of individual metabolites and the levels of confidence we have in those identifications, presented in this manuscript. We outline our responses to each individual question below, but we would like to first note several concepts and issues associated with generation and analysis of these types of data that may aid in answering the reviewer's questions.

Metabolite identification from LC-MS experiments was carried out by matching accurate masses and retention times (RTs) of authentic standards (MSI confidence level 1: an annotated compound that was matched to a standard with two orthogonal approaches). However, it is beyond the scope of any study to provide authenticated standards to many hundreds of detected compounds/peaks, hence full datasets are deposited in public repositories (i.e. MetaboLights) in the spirit of open access data. In the case of our experiments, when authentic standards were not available, predicted retention times were calculated by a previously validated model (PMID: 21928819; MSI confidence level 2: a putatively annotated compound based on its exact mass determination). Published literature and pathway/genome databases were considered to improve annotation in cases where isomers could not be differentiated based on accurate mass and retention times. Many of the metabolite names given by the Ideom software used in this analysis are generated automatically, as the software provides a best match to database entries of the given mass and formula. In the absence of additional information, these must be considered as putatively-annotated hits and the confidence scores adjacent to the metabolite ID serves as a guide to this.

With regards to identification of lipids, as these are largely hydrophobic, they are generally not resolved by ZIC-pHILIC chromatography and undergo large amounts of ion suppression as they mostly elute together at the very start of sample runs, making ID by retention time challenging. Furthermore, the use of mass (m/z) alone for lipid identification is problematic due to the fact that it is impossible to identify the locations of one or more double bonds in unsaturated fatty acids/lipids. Therefore, Ideom provides a list of possible isomers for putative identification of the lipid species. To clarify our processes around this, we have amended the methods section by the addition of more detailed explanation of the processes involved in metabolite identification (lines 1132-1152).

We note that of the 207 identified metabolites in the supernatant metabolomics experiment, 100 of these are putatively annotated as lipids, although again, these are not discussed in great detail due to the outlined complexities in metabolite identification. For clarification of this aspect, we have also inserted a line into the main text (lines 239-242), which reads as follows "Of the 207 metabolites in the final dataset, 100 were putatively annotated as lipids. Annotations of putative mono- and poly-unsaturated fatty acids are likely of lower confidence due to the challenges of identifying these metabolite species using this LC-MS platform (see methods for details)". There is higher confidence in the identification of fully saturated fatty acids, such as those discussed in figure 7, for the reasons discussed above (fewer isomers, and better resolution of mass and retention time). We have therefore kept these results in the manuscript.

Furthermore, we have revised the supernatant metabolomics supplementary table, with appropriate annotation to help clarify the confidence level for each metabolite identification. Each putatively identified metabolite has been formatted with a drop-down list of potential isomers. We note that the confidence level (out of ten) given in column "F" refers to the metabolite with the highest confidence match, and we have added conditional formatting to strike-through this score when an alternative isomer is selected. We have added further explanation to the supplementary data legend, and we hope these changes will aid interpretation of and navigation through this dataset.

There are several issues about metabolic identity. i.e. Table 2 what is dactyl and trihydroxy-fatty acids (C18:1) is not a realistic assignment. Fig 2B, unsure how the authors know that it is "12"-hydroxydodecanoic acid, as this is not a standard.

The metabolites annotated as diacetyl (7 isomers) and 12-hydroxydodecanoic acid (15 isomers) are automatically generated putatively annotations based on mass determination. As these metabolites were putatively identified based on mass and retention time matching public databases, we have decided to include them in the results overview in the interest of open science. However, as we now include a statement in the methods section on the tentative identity attributed to any metabolites – we believe that investigators who might look to this manuscript, for example should a metabolite of the same mass appear in a future dataset, but due to a particular outstanding biological role, warrant further structural characterisation and turn out to be different from the tentative assignment here, then it would be clear to those investigators that the same metabolite was identified here, but given a tentative assignment based on information available at the time which can be superseded with information available from further work. Nevertheless, even with a matching mass, it is difficult to determine which position is hydroxylated in the putatively identified 12-hydroxydodecanoic acid. We have therefore removed the position, leaving the putative identification as "hydroxydodecanoic acid" (Fig 2 and S3 table).

Fig 2 Does the total amount of choline, choline-phosphate, glycerol-P-choline observed in the media correlate with the amount of lyso-PC that has been degraded.

The supernatant metabolomics experiments did not include standards for peaks putatively identified as Lyso-PC. In addition, this experiment did not include steps for absolute quantification of metabolites, and given the fact that mass spectrometry peak areas are a function of absolute quantity with ionizability and potential ionizability-competition with co-eluting metabolites, such quantification is beyond the scope of this work. Such an approach is only typically included in targeted metabolomics experiments. Therefore, we can only interpret these results based on relative changes in peaks. There are several putatively identified fatty acids carrying PC moieties that degrade in supernatant samples over time (e.g. LysoPC(17:0), LysoPC(18:1), PC(15:0), PC(16:0) and PC(16:1)). It is therefore very difficult to establish the origin of the choline, choline phosphate and sn-glycero-phosphocholine build-up in the media. As we are unsure of the exact identities of these fatty acids, we have not described them in detail in this manuscript, but this is an aspect of T. congolense metabolism that we are very interested in investigating further. We have added a sentence to the manuscript (lines 275-277) that reads as follows: "However, given the putative nature of these fatty acid annotations, we could not establish the origin of the elevated choline-related metabolites."

Fig 2: Why is there inosine 1 and 2?

Two peaks corresponding to inosine were detected in the supernatant experiment. We have investigated this further and established the genuine inosine peak based on the authentic standard, peak intensity, mass and retention time. We have therefore re-assigned the secondary peak as "inosine-related". We believe this second peak is likely an isomer, or alternatively, a metabolite that has fragmented to provide inosine (e.g. IMP). However, without further study (which is beyond the scope of this manuscript) we cannot confirm this at this time. We have revised Fig 2 and S3 table to reflect the above.

Fig 2B Lyso PC(17:0), not of eukaryotic origin to any great extent, but this is not in Fig 2A??
Very concerned that the authors find some many aldehyde containing metabolite.

The peak annotated as LysoPC(17:0) was not included in Fig 2A as this metabolite did not change significantly in the T. congolense supernatant analysis (FDR = 0.1357; this can be seen in Fig 2B – scatter dot number 15 is highly reduced in the T. brucei dataset – taken from PMID 23571546 – only).

In regards to aldehyde-containing metabolites in the data overview, as stated in responses to other comments, annotations of metabolites are based on IDEOM's output. We have presented this output (S3 table) taking IDEOM's top listed metabolite names matched from the searched databases to a given mass, but we are by no means suggesting that we believe the detected feature is definitely that metabolite. It is not possible to absolutely determine identities in the absence of authentic standards. In some instances, there are no doubt more probably annotations to assign, but as this is purely speculative, we elected to list IDEOM's output, whilst including potentially isomers, detected masses and detected retention times, so that prospective users of the data can make their own judgements.

Fig 5: can the authors say anything about cytosine, as T. brucei do not take up it up, do you know if T.co does?

Whilst a peak matching the monoisotopic mass of cytosine was identified in the supernatant metabolomics dataset, the peak retention time (RT) was 6.5% outside the RT window of the authentic standard, and the identification was therefore rejected (maximum allowed RT discrepancy was set to 5%). The intensity of this peak did not change significantly over time, indicating there was no exhaustion or accumulation of this metabolite. However, given the RT discrepancy, we cannot definitively state whether a) this peak corresponds to cytosine, or b) T. congolense has a capacity for cytosine uptake. A previous study (PMID: 23556014) has noted that T. congolense has an expanded nucleobase transporter repertoire, and we agree with the reviewer that it would therefore be of interest to find out which nucleobases this species sources exogenously as opposed to de novo biosynthesis.

Unfortunately, cytosine and its derivatives were not detected in the ^{13}C-D-glucose labelling dataset using our default pipeline, and we are currently assessing whether these data may still harbour clues as to whether labelled cytosine can be deconvoluted, as labelled cytosine was detected in T. brucei intracellular samples after incubation with ^{13}C-D-glucose (PMID: 725775470 – note that this study used slightly different LC-MS methodology). We have added several sentences to the discussion section of the paper (lines 840-844) which reads as follows: "This study was not able to assess whether T. congolense has a capacity for cytosine uptake. It is established that T. brucei does not take up this pyrimidine [121], but given the expanded nucleobase transporter repertoire in the T. congolense genome [21], it would be of interest to carry out more in depth analysis of cytosine metabolism, as well as nucleotide metabolism in general, given the interest in drug development."

Fig 6 can the authors comment on deamination or decarboxylation of amino acids, other then glutamine/glutamate and Leucine, how about some of the aromatic amino acids Tryptophan?

There are a lack of ref for some of the comment in the amino acid metabolism section, i.e. serine and cya and trypanothione.

Whilst the stable isotope labelling data (S8 Fig) gives us some idea as to carbon usage (and therefore, some idea of decarboxylation) from selected amino acids, it was outside the scope of this study to investigate metabolism of every amino acid in detail, and we hope the data generated in this study can be used by other groups for this purpose. With regards to aromatic amino acids, based on amino acid exclusion studies, only tyrosine appeared to be essential (likely because the goat serum supplement contained sufficient amounts of tryptophan and phenylalanine). In T. brucei, deamination of the three aromatic amino acids is carried out by the cytosolic aspartate aminotransferase, abundance of which is unchanged between the species (adjusted P value = 0.289; S1 Table). There is currently no data to confirm the same process occurs in T. congolense and in addition, there is increased abundance of mitochondrial asparate aminotransferase in culture-derived T. congolense, the importance of which has not been investigated further. Kynureninase, another enzyme involved in tryptophan degradation, is unchanged in ex vivo samples, but decreased in culture-derived T. congolense, compared to T. brucei. We agree tryptophan, in particular, is an important amino acid to trypanosomatids, and its role in T. congolense metabolism should be investigated more in depth.

We apologise for the lack of referencing in the sections mentioned by the reviewer, and have tried to amend this by adding in references where applicable.

It is very interesting that fatty acid synthesis is not observed from Glc/Thr. Do we know if the GPI anchors in T.co still utilise Mys

We could only find one previous study investigating GPI anchors in T. congolense (PMID: 2957588), which concluded that, like in T. brucei, glycosyl-sn-1,2-dimyristylphosphatidylinositol is the GPI anchor, suggesting that myristic acid is still a major requirement for this species. We have added a sentence to the manuscript to discuss this (lines 846-848). We agree that the result from labelled Glc/Thr studies with regards to fatty acid synthesis was an unexpected one, and are hoping to carry out more work identify how T. congolense sources the large amounts of myristic acid (and other fatty acids) it presumably requires – albeit beyond the scope of this manuscript. This work will be carried out with protocols developed specifically for the analysis of lipids and fatty acids.

Was lipoic acid observed in the analysis,as this is always de novo synthesised by kinetoplastids? Did the authors observe any label from Glc or The into the mevalonate pathway, not necessarily sterol, but farsenyl and other isoprene products. As there are difference in the expression levels of these enzymes HMGCR, IDI, MDD etc?

Lipoic acid was not identified in experiments carried out in this study, as not all metabolites are compatible with the mass spectrometry system (LC-MS) used. In previous studies, this metabolite is typically detected by specific bioassays (e.g. PMID: 1501642, 17166831). However, we further analysed our RNAseq data to look for genes involved in the synthesis of lipoic acid. For lipoic acid synthase (TbTc_4472), orthoTPM values were very similar between T. brucei and T. congolense (log_2 fold change of 0.06 and 0.36 for ex vivo and in vitro, respectively, p = 0.115). A similar pattern was seen for two annotated copies of dihydrolipoamide dehydrogenase (TbTc_0275 & TbTc_0276). Given that genes for lipoic acid de novo synthesis are present in T. congolense, and their expression is unchanged, we presume that lipoic acid serves a similar role in T. congolense. We have added text to the manuscript (lines 624-628) to discuss these results.

With regards to the pathways mentioned by the reviewer, we did not specifically look at the mevalonate/isoprenoid biosynthesis pathway in the metabolomics data for the reasons outlined above. However, we re-analysed the mzMatch-ISO datasets that were generated for these studies to analyse ^{13}C-carbon labelling, in order to try to answer the reviewer's question. Specifically, we searched for metabolites related to farnesyl, squalene, mevalonate, isopentenyl diphosphate, geranyl diphosphate, lanosterol and ergosterol. Unfortunately, the only peaks found were a

putative peak for mevalonate (glucose labelled data set, no carbon labelling was observed), and therefore there is insufficient evidence in our data to warrant discussing this in the manuscript.

We note that several co-authors on this manuscript have carried out a comparative lipidomics study of T. brucei and T. congolense, using techniques specifically designed for the analysis of lipid and fatty acid species. These results are currently being analysed and prepared for submission, and we hope this study will help to answer some of the reviewer's interesting questions in more detail.

Do the authors think it is worth looking at the SHAM Ec50 in the presence of higher glycerol concentration, as this in Tb is affected?

This experiment was a very interesting suggestion by the reviewer, and we have therefore carried out further drug sensitivity assays with SHAM in the presence of higher glycerol concentrations in both species (we used a glycerol concentration of 10 mM based on a previous study – PMID: 30383867). The results of this analysis have been included in Table 1. Whilst EC_{50} values for SHAM alone are higher than previously recorded, the difference between the species remains significant. In addition, addition of 10 mM glycerol appears to increase sensitivity to SHAM in both species by ~12-13-fold.

We note that Pineda and colleagues carried out important work on glycerol as a carbon substrate for T. brucei. This study involved adaptation of cells over the period of a month, to medium that was depleted of glucose, but contained either 10 mM glycerol or a combination of 10 mM glycerol and 50 mM N-acetyl-D-glucosamine. Cells adapted to the glycerol-based media were 11.5 times more sensitive to SHAM in this study (compared to 12.1 times more sensitive in this study). A similar study on T. congolense would be of interest, but given the time involved, we feel it is outside the scope of this manuscript.

Part III – Minor Issues: Editorial and Data Presentation Modifications

Reviewer #1:

1- In lines 191-195, it is mentioned that some metabolic genes are not present in the T. congolense gene, but it would be informative to show the syntenic maps of these areas between T. brucei and T. congolense to illustrate the possible gene deletion in the T. congolense genome (supplementary data), and are metabolic genes absent in the T. brucei genome, compared to the T. congolense genome? For example, it is not mention in this list that MPC1 is missing in the T. congolense genome. This synteny-based analysis would be helpful to confirm the gene deletion.

We thank the reviewer for this suggestion, and based on this, we carried out synteny-based analysis to further assess how likely the gene deletions are. Interestingly, we found that for MPC1, delta-4 desaturase and SDH11, the deletions appear to be genuine, as surrounding regions are highly syntenic. However, in the case of guanine deaminase, the locus appears to be subtelomeric in T. brucei. These regions are divergent between T. congolense and T. brucei and therefore, synteny-based analysis is difficult. We can therefore only conclude that, based on the current assembly of the T. congolense genome, guanine deaminase has been lost, although improvements in assembly of the subtelomeric regions may shed more light on this.

We have generated a new supplementary figure (S1 fig) showing the synteny-based analysis for SDH11, MPC1 and delta-4 desaturase, and revised the relevant portions of the manuscript to outline the above. We have rewritten the paragraph addressing synteny (lines 198-209).

2- In Figure 2B, the metabolite names are shown on the figure and in the figure legend. This is redundant.

This has been amended, we have removed the metabolite names from the figure legend, leaving them in the figure itself.

3- In Figure 3, for convenience it would be helpful for non-specialists to include in Fig 3I the step numbers of Fig 3G. It would also be useful to indicate the FBPase step in Fig 3G, for instance by 3b (3a been phosphofructokinase).

Thank you to the reviewer for this suggestion. We have amended the figure to include numbers in brackets in Fig 3I that correspond to the numbers in Fig 3G.

The figure legend mentions that "typically, the succinate shunt is only active in PCF T. brucei, with low levels of activity in BSF T. brucei". If low levels of activity of the succinate shunt have been described in BSF T. brucei, it is not ONLY active in PCF T. brucei. This should be rephrased.

We apologise for this imprecise text. We have removed this sentence from the figure legend as we do not believe it is needed, and succinate shunt activity is already discussed in the main text of the manuscript (lines 308-318).

There is no glucose transporter in the glycosomal membrane since glucose is exchanged between the cytosolic and glycosomal compartment through a non-specific channels. Thus, the number 1 in Panel B has to be removed. In addition, step 12 should be removed since it PEP exchange between the cyotosolic and glycosomal compartments through the same non-specific channels. Thus, PEPCK corresponds to step 12, etc. until production of alanine and acetate.

We thank the reviewer for bringing this to our attention, and have amended Fig 3G. Upon further analysis we found we had also missed the enolase step (3-PGA → 2-PGA). We have further amended the pathway figure to include this step and adjusted the numbering accordingly.

4- In pages 13-14, there are some redundancy between lines 307-308 and 333-340, such as "to confirm that the elevated levels of succinate and malate seen in T. congolense spent medium samples originated from glucose ..." (lines 307-308) and "to determine whether the elevated succinate in supernatants originated from glucose catabolism, metabolite labelling was corrected …" (lines 333-334). This part can be shortened.

We have shortened this text in order to remove redundancy (new lines: 319-321).

5- Lines 334-336 describes how the authors corrected the metabolite labelling in order to compare with the theoretical 1:1 (50%) ratio of natural glucose to 13C-U-D-glucose. Since, this correction is meant to get a 50% of 13C-U-D-glucose (instead of 43.1%), how >90% labelling in glycolytic metabolites up to pyruvate can be obtained, since it should not exceed 50%? Theoretically, these values should not exceed 50%. This, it seems that 100% labelling of glucose has been considered as a reference (instead of 50%) to analyse these data set. This is not correct. The authors should use as a reference 50% of labelling for glucose and correct the values of other metabolites accordingly.

Thanks to the reviewer for spotting this error. We initially corrected for 100% labelling in order to compare our results to an analogous study of ^{13}*C-D-glucose carried out in T. brucei stable isotope study (PMID: 25775470), but have now corrected for 50% labelling instead. In addition, we have revised the raw labelling percentages by including fractions with >2 carbon labels (we had previously only included >3 carbon labels).*

6- Line 349, ref 37 should be included with ref 35.

This has been amended, due to revisions, new reference numbers are 38 and 40.

7- The sentence lines 349-351 is confusing "similarly, no citric acid cycle intermediate isotopologues (e.g. citrate) were found when BSF T. congolense were incubated with 13C-U-D-glucose, although small amounts of 2-carbon labelled succinate and malate were observed (Fig 3H). Does it mean that 2-carbon labelled succinate and malate could be generated through the TCA cycle activity? Actually, in trypanosomes, 2-carbon labelling of succinate and malate (instead of 3-carbon labelled) is mainly due to the reversibility of the glycosomal succinate branch (from PEP to fumarate) and the symmetry of the fumarate molecule, which makes carbons 1 and 4 indistinguishable by fumarase. Indeed the reverse reaction catalysed by PEPCK (decarboxylation of malate to PEP), can randomly decarboxylates carbons 1 or 4 and thus remove 13C-carbons (50%), which is then replaced by a non-13C-enriched carbon through the forward PEPCK reaction. Thus, production of 2-carbon labelled succinate and malate can easily be explained by the reversion of the glycosomal succinate branch without invoking a possible TCA cycle activity.

We have rewritten this paragraph taking into account the reviewer's comments. We hope this clarifies the results, whilst taking on board what the reviewer has stated (new lines: 361-363).

8- Lines 389-390, in the presence of glucose as performed all along the manuscript, PPDK is not an enzyme of the succinate shunt since it is used to produce pyruvate from PEP. Consequently, this RNAi analysis is not relevant to the question asked. In addition, the low efficiency of the RNAi approach to knockdown PPDK (50%) does not allow a conclusion as to the role of PPDK. This experiment should be removed from the manuscript.

We accept the reviewer's point, and have removed the RNAi experiment from figure 4, and removed all references to the PPDK RNAi experiment from the manuscript.

9- In the same line, RNAi-mediated knockdown of PEPCK is inconclusive for the same reasons, since 50% of the mRNA remains after induction. Instead of measuring the transcript levels, it would be more relevant to measure the PEPCK activity or effect of PEPCK partial knockdown on succinate production from metabolism of glucose. Anyway, this experiment should also be removed from the manuscript.

Likewise, we have removed the PEPCK RNAi experiment and all references to it from the manuscript. As a result, we have now moved S2 Fig (T. congolense growth in GlcNAc) into Fig 4, which now includes growth of T. congolense in reduced glucose, 2DG, GlcNAc and the HT RNAi experimental data.

10- In figure 7, the ACS abbreviation has been used both for acetyl-CoA synthetase and acyl-CoA synthetase. To avoid confusion acetyl-CoA synthetase should be abbreviated AceCS as previously done. In line 1281, T. congolense should be in italics.

These have both been amended.

11- Line 414, remove the "s" from alternatives.

This has been amended.

12- In line 415 and 618-619, the authors refer to Trindade et al. 2016 (ref 72) to support the *T. brucei's* use of b-oxidation for energy production. In fact, this work doesn't show it and the use of fatty acids for ATP production has not been demonstrated in *T. brucei* so far. Ref 72 showed that myristate (C14 FA), which is not myristyl-CoA (substrate for b-oxidation), can be reduced, hydrated and then reduced once more. However, production of C12 FA (or shorter FA), with the expected production of acetyl-CoA required for energy production, has not been observed. Actually, Trindade *et al.* did not conclude that BSF produce ATP from fatty acid catabolism. Thus, the author should not used ref 12 to support their hypothesis regarding the role of fatty acids in energy production of *T. congolense*.

We have removed referencing to Trindade et al. from the manuscript and we agree with the reviewer that fatty acid oxidation has not been shown in T. brucei. Given that this study only compared T. congolense to T. brucei, we did not think it was justifiable to include references to other trypanosomatids with regards to fatty acid oxidation, and have therefore toned down discussion of this metabolic pathway, limiting this to say that it is important to establish whether T. congolense has a capacity for FA-oxidation.

13- In lines 418-419 is stated that "BSF T. brucei do not detectably express any ETC components with the exception of the reversed F1Fo-ATPase and alternative oxidase". Actually, one co-author of this manuscript published in Ref 92 evidences that complex I of the respiratory chain is expressed and functional in BSF T. brucei. These data should be taken into account and included in the discussion of the ETC components, including complex I.

We have revised this paragraph (lines 411-418), which now reads as follows: "PCF T. brucei express most components of the electron transport chain (ETC) to generate ATP through oxidative phosphorylation. In contrast, BSF T. brucei express an F_1F_o-ATPase that functions in reverse, and alternative oxidase [76]. In addition, a recent study has suggested that complex I is expressed and functional in BSF T. brucei [77]. Transcriptomics analysis of the ETC was carried out, using a gene list generated by Zikova and colleagues [76], but no significant patterns could be discerned, and thus we were not able to draw a conclusion with regards to ETC activity in BSF T. congolense based on transcriptomics data alone (S1 Table, S3 Fig)."

14- Line 453, to help the reader it would be good to include in the text the abbreviation of hypoxanthine-guanine phosphoribosyltransferase and uracil phosphoribosyltransferase used in the corresponding figure.

This has been amended, and we have added abbreviations where applicable.

15- Lines 601-602 (and Figure 7C), Since ACH has been mentioned in this section, it would be relevant to include data for the ASCT (TbTc_0236), which is also involved in acetate production from acetyl-CoA, but coupled with ATP production.

We have revised the results paragraph that mentions ACH (and corrected "thioesterase" to "hydrolase" as indicated in the TREU 927 annotations), and added a sentence on ASCT, which is expressed at higher levels in T. congolense compared to T. brucei (this can be seen in Fig 8). These sentences now read as follows (lines 606-611): "…ASCT (TbTc_0236), which catalyses ATP-coupled acetate production, was higher under both conditions in T. congolense (log2 fold changes: 2.04 and 0.46 for in vitro and ex vivo, respectively; $p < 0.001$; S1 Table). Conversely, acetyl-CoA hydrolase (also known as acetyl-CoA thioesterase, ACH; TbTc_5515), an enzyme involved in ATP synthesis-uncoupled acetate production in T. brucei [100] was expressed at lower levels in ex vivo T. congolense compared to T. brucei (Fig 7C)."

16- Line 683, "was" can be replaced by "were".

This has been amended.

17- Line 732, what about Fumarate reductase (FRDg), which is the last step of this succinate-producing shunt?

We have added a sentence (lines 749-751) explaining that, whilst most components of the succinate shunt were expressed at higher levels, fumarate reductase did not fit this trend, for reasons that we cannot identify.

18- Lines 784-788, the authors misunderstood the structure of the mitochondrial pyruvate carrier (MPC) composed of two small hydrophobic paralogous proteins, MPC1 and MPC2, which are essential and sufficient for the transport of pyruvate into mitochondria. Thus, MPC is composed of

MPC1 plus MPC2, meaning that the absence of MPC1 in the *T. congolense* genome should be considered as a problem. The authors wrongly considered that *T. brucei* contains 2 MPC transporters (MPC1 and MPC2), while *T. congolense* a single one (MPC2). Consequently, they have to reconsider their analysis of this unexpected situation in *T. congolense*, since the production of acetate from glucose implies the existence of a functional mitochondrial pyruvate carrier.

The synteny analysis mentioned above (see S1 Fig) indicates that MPC1 is not encoded in this region of the genome on T. congolense. Moreover, MPC1 is not annotated anywhere else in the T. congolense genome based on analysis using both orthMCL (TriTrypDB) and orthofinder (this study). Given the nature of the Pacbio assembly of the T. congolense IL3000 2019 genome construction, there is a high probability that MPC1 is not encoded in the T. congolense genome (although we cannot rule this out definitively as there are still some unassembled contigs, some of which are likely to be subtelomeres that significantly diverge from T. brucei). Furthermore, a previous study suggested that there is likely another mitochondrial pyruvate carrier that is insensitive to UK5099 (PMID: 26748989).

We have rewritten the lines discussing mitochondrial pyruvate transport to take into account the reviewer's comments which reads as follows: "Interestingly, T. congolense does not appear to encode a syntenic orthologue of MPC1 (S1 Fig), and attempts to identify the gene by orthoMCL or Orthofinder were unsuccessful. In T. brucei, the mitochondrial pyruvate carrier is composed of two paralogous proteins, MPC1 and MPC2, both of which are essential [104]. The absence of an MPC1 orthologue in T. congolense, in spite of these cells requiring mitochondrial pyruvate transport, inferred from acetate production from glucose, indicates a key difference in the constitution of pyruvate carrier in this species. Structural differences to pyruvate carriers between the two species, or the enhanced mitochondrial pyruvate catabolism in T. congolense may explain its enhanced sensitivity to UK5099, a mitochondrial pyruvate transport inhibitor. In addition, previous data suggest that in T. brucei, there is likely another mitochondrial pyruvate carrier that is insensitive to UK5099 [104]" (lines 788-799).

19- In line 835, it has not been demonstrated that the putative T. brucei CL gene codes for a CL. Thus, it would be better to replace "this gene" by "this putative CL gene".

We agree with this comment and have amended the text as suggested.

Reviewer #2

Reviewer #2: First, this reviewer, who is not native in English, feels uncomfortable by pointing the many mistakes in the use of this language. Some of them are pointed in the annotated pdf attached. Of course the Reviewer is aware that he/she can be wrong about some of the pointed mistakes but he/she is sure that the MS must be revised. About the style, the authors chose to produce a MS in which Results and Discussion are separated sections, however most of the discussion is made in the Results section. As the MS "touches" many different aspects of metabolism and makes many comparisons and links with the literature, this option is understandable, but makes the Discussion section highly repetitive, with little addition of information and/or ideas. I suggest to maintain the structure but shortening considerably the Discussion and limiting it to the aspects that are not discussed in Results. For example, the authors could privilege a more integrated analysis of the obtained data. This is just a suggestion and the authors should not consider this mandatory. Something else that the authors should reconsider is the claim that the article "highlights potential areas that are exploitable for pharmacological intervention". In fact, from the amazing quantity of information offered to the reader there are little clues on new points of intervention with drugs. In the opinion of this reviewer this claim should be touched down.

We have addressed all the comments from the annotated pdf file provided by the reviewer individually below. We thank the reviewer for pointing out the mistakes. We have gone over the

manuscript carefully and tried to shorten the discussion, as suggested by the reviewer. The suggestion of the reviewer that we change the emphasis away from identification of drug targets is similar to the point made by the editor (see detailed response above), and we have amended the manuscript title and text accordingly.

Line 26, the boodstream stage of

This has been amended.

Line 30, Compared with what? Which is the link between transcriptomics and activities (I suppose the authors refer to enxymatic activities?)?

We apologise for the lack of clarity. This has been amended, the sentence now reads as follows: "Transcriptomics analysis showed higher levels of transcripts associated with the mitochondrial pyruvate dehydrogenase complex, acetate generation, and the glycosomal succinate shunt in T. congolense, compared to T. brucei."

Line 33, this sentence is weird

We apologise to the reviewer. This sentence has now been deleted from the revised abstract.

Line 37, Which is the meaning of "essential" in this context? If the cell is able to survive when something is absent, this thing cannot be considered essential.

We apologise for lack of clarity. We have amended this sentence to suggest that, based on our findings, electron transport chain activity is not an essential process in BSF T. congolense. The sentence now reads as follows (lines 35-38): "To validate the metabolic similarities and differences, both species were treated with metabolic inhibitors, confirming that electron transport chain activity is not essential in T. congolense. However, the parasite exhibits increased sensitivity to inhibition of mitochondrial pyruvate import, compared to T. brucei."

Line 38, Increased dependence on pyruvate mitochondrial uptake?

We feel that mitochondrial pyruvate import is more apt in this context.

Line 39, higher? increased?

This has been amended to "higher."

Line 48, You already used the complete name of species and then the abbreviated name, so continue the same. Replace for *T. congolense*.

We have aimed to adhere to the PLOS Pathogens guidelines with regards to referencing species names: "Write in italics (e.g., Homo sapiens). Write out in full the genus and species, both in the title of the manuscript and at the first mention of an organism in a paper. After first mention, the first letter of the genus name followed by the full species name may be used (e.g., H. sapiens)" – Our interpretation of these guidelines is that we should write the full species name at first mention in all of the abstract, authors summary and manuscript body.

Line 63, metabolic areas

This has been amended. The instance of "potential areas" has been removed in order to change the emphasis away from drug target identification. The sentence now reads as follows: "Our study provides a foundation of functional metabolic knowledge on T. congolense, with insights into how this parasite fundamentally differs from T. brucei."

Line 66, Again: T. congolense.

As this is the first instance of the species in the main text of the manuscript (i.e. after abstract and author summary), we have decided to leave this as "Trypanosoma congolense."

Line 68, too much repetition of AAT in this paragraph, two times in a single sentence for example.

We agree with the reviewer. We have amended the paragraph and we have removed three instances of "AAT". The paragraph now reads as follows (lines 66-76): "The hemoflagellate protozoan parasite Trypanosoma congolense is a primary causative agent of animal African trypanosomiasis (AAT), which can also be caused by T. vivax and T. brucei [1]. AAT is one of the most important livestock diseases across sub-Saharan Africa and accounts for livestock deaths in excess of 3 million annually, with up to 120 million cattle at risk [2-4]. Current methods of disease control centre around chemotherapy and prophylaxis (reviewed in [3]), but the very few available veterinary trypanocidal drugs have been used extensively for decades, resulting in resistance and inadequate protection [5-8]. In contrast to T. brucei [9], the resistance mechanisms of T. congolense are still poorly understood [10]. As such, there is a critical need for the development of new and improved chemotherapeutics to manage AAT [3, 11], and furthering our knowledge of how T. congolense develops resistance to drugs can facilitate optimising the lifetime of both existing and new drugs."

Line 73, direct

We acknowledge that "dire" is not the best adjective to use, and have exchanged this for "critical". The sentence now reads: "As such, there is a critical need for the development of new and improved chemotherapeutics to manage AAT,…" (lines 73-75).

Line 76, which groups subspecies T. B. gambiense and T. b. rodhensiense causing...

This has been amended with a variation, the sentence now reads as follows: "Most of our biological understanding of African trypanosomes derives from studies on T. brucei, subspecies of which (T. b. gambiense and T. b. rhodesiense) cause Human African Trypanosomiasis (HAT) [12]" (lines 77-79).

Line 79, at

This has been amended.

Line 86, There are specific genes that are different or the authors refer to the genome structure, expression profile, presence/absence of certain genes?

We apologise to the reviewer, we originally did not include further elaboration in the interest of manuscript length, but have added in an example of gene differences based on a previous study (PMID: 23556014; lines 89-91). In this case, we are referring to differences in genome content. As the reviewer alludes to, we have also found examples of specific genes missing from the most up-to-date genome assembly of T. congolense IL3000, which we describe in the results section (lines 198-209).

Line 211, Comparative metabolite consumption and output between T. congolense and T. brucei

This portion of the manuscript is heavily focused on the metabolic inputs and outputs of T. congolense alone, with only a short paragraph comparing these data to a published T. brucei dataset. We therefore feel the current heading is suitable.

Line 275, Maybe this sentence can be the beginning of the next paragraph.

This has been amended as requested by the reviewer.

Line 291, when comparing different species, the terms "upregulated" or "downregulated" should be avoided. I suggest to use more/less expressed. Regulation should refer to a cell or population of cells that changes the expression level(s) of (a) gene(s(as a response to environmental/internal changes.

We agree, and thank the reviewer for raising this point. We have amended instances of "upregulated" (3×) to "expressed at higher levels", but could not find any instances of usage of "downregulated."

Line 295, But not in…

This has been amended.

Line 309, what are exactly "cell pellet extracts"?

We apologise for lack of clarity. Cell pellet extracts are metabolites isolated from centrifuged cells. This includes washing steps to ensure that no supernatant is carried over into these metabolite samples. We appreciate we did not word this well, and have amended this sentence. The sentence now reads as follows (lines 319-321): "To test whether elevated levels of succinate and malate seen in T. congolense supernatant medium samples originated from glucose, LC-MS analysis using ^{13}C-U-D-glucose was carried out on intracellular metabolites isolated from in vitro-cultured cells."

Line 319, matched those that would be expected...

This has been amended, and the sentence now reads as follows (lines 328-330): "Labelling ratios of downstream metabolites were largely similar to that of intracellular glucose, and carbon labelling patterns matched those that would be expected in the BSF T. brucei glycolytic pathway."

Line 329, Not necessarily the formation of Acetyl-CoA implies the production of Acetate, since Acetyl-CoA can be "fully" (in fact majoritarily) used in other processes.

We apologise for lack of clarity in this statement. We agree with the reviewer's comment that acetyl-CoA is used by many intracellular processes, including, for example, fatty acid biosynthesis (PMID: 17331998). We have toned down the sentence, which now reads as follows (lines 336-340): "Although acetyl-CoA, the product of pyruvate oxidation, was not detected for technical reasons, the 2-carbon labelling patterns of acetylated metabolites suggests that the acetyl moieties in these compounds originate from glucose, potentially through acetyl-CoA and acetate, as previously evidenced in other trypanosomatids."

Line 386, This experiment is not conclusive at all. 2DG is a competitive inhibition of hexokinase, which in principle arrests glycolisis, but it has another systemic effect which is many often not taken into account: it produces a drastic change in the ATP/ADP ratio due to a rapid consumption of ATP in the "upper side of glycolisis" whout the "payoff". Therefore, this results could be due to a "crash" of the ATP levels, speciffically to the inhibition of glycolysis, or (more likely) to both.

We agree with the reviewer that 2DG is an inhibitor of hexokinase that also results in an arrest of glycolysis, as well as inhibition of the pentose phosphate pathway. In addition, production of 2-deoxy-D-glucose 6-phosphate consumes ATP that is not regenerated in the latter stages of glycolysis, leading to glycosomal ATP depletion.

However, given the nature of this study, which aimed to generate a comprehensive overview of T. congolense metabolism, it was still crucial to establish whether the effects of 2DG-mediated inhibition were lethal for T. congolense. If ATP-generating pathways other than glycolysis exist, it would be hypothesised that these could compensate (even if the glycosomal membrane is impermeable to ATP/ADP). Therefore, this experiment shows us that in T. congolense, most, if not all ATP originates from the catabolism of glucose, similar to BSF T. brucei. We therefore feel

that, even though the result is not particularly exciting, it is still an important one in terms of defining T. congolense metabolism.

With the reviewer's valid comments in mind, we have revised some of the wording in the relevant sections. This reads as follows (lines 374-380): "To test whether glucose-derived ATP was essential to T. congolense, cells were incubated with D-glucose in addition to varying concentrations of 2-deoxy-D-glucose (2DG), which can be internalised, but not metabolised further than 2-deoxy-D-glucose 6-phosphate. As a result, glycolysis and pentose phosphate pathway metabolism are inhibited and glycosomal ATP levels are depleted as they cannot be regenerated in the latter stages of glycolysis, leading to a drastic change in glycosomal ATP/ADP ratio [72] (Fig 4B)."

Line 378, The authors should be aware that, if hexokinase is able to phosphorylate GlcNAc into GlcNAc-6-P (as observed by (Yamada-Okabe et al., 2001; Rao et al., 2013 among others), this metabolite can feed glycolysis at the level of Fructose-6-P which would change the interpretation of these results.

We note the references given by the reviewer are primarily studies in Candida albicans, a fungal pathogen unrelated to African trypanosomes. Supplementation of in vitro culture with GlcNAc has frequently been used as a method of starving both PCF and BSF T. brucei of glucose (PMIDs: 18371239, 30383867). Ebikeme and colleagues (PMID: 18371239) showed that GlcNAc cannot be used as a direct energy source by T. brucei and importantly, the metabolite is not internalised by these pathogens. Instead, GlcNAc binds hexose transporters, effectively blocking glucose uptake. We feel that, given the similarity of glucose transporters between T. congolense and T. brucei (sequence similarity of 78-84%; PMID: 10903520), and the fact these are related species of protozoa, that GlcNAc would likely have a similar effect on BSF (and PCF) T. congolense.

Line 408, How this fits to the findings published by Haanstra et al., 2017 "Targeting pathogen metabolism without collateral damage to the host" publishes in Sci. Reports?

Using in silico modelling, Haanstra and colleagues showed that it is possible to kill a pathogen like T. brucei selectively even when targeting a pathway essential to both host and pathogen. Notably, T. brucei exhibit very high flux through the glycolytic pathway and 50% inhibition is enough to kill the parasite. T. congolense, as we have evidenced, exhibits a much lower flux and, whilst we cannot be sure without further experimental evidence, we believe that 50% inhibition would not be sufficient to kill T. congolense. RNAi penetrance is not ideal in T. congolense, yet 40-50% inhibition is attainable, and cells remained viable under induced glucose transporter RNAi conditions.

This was a very interesting question posed by the reviewer, and we have included a sentence in the discussion to acknowledge this (lines 739-740). The sentence reads as follows: "This reduced flux indicates that T. congolense is unlikely to be as susceptible to glycolytic inhibition as T. brucei, where 50% inhibition is sufficient to kill the parasite [27]."

Line 455, Theories or findings?

We apologise for the lack of clarity in this sentence. This has been amended to "suggestion", as the evidence was based on genome content only (and has as-of-yet not been confirmed by further in vitro experiments in T. congolense). In addition, we have also amended "upon" to "on". The sentence now reads as follows: "Upregulation of nucleoside hydrolases and phosphoribosyltransferases supports a previous suggestion based on genome content that T. congolense has a capacity for nucleobase uptake."

Line 505, Is glutamine synthase present in T. congolense? If so, is Gln essential? Other Gln biosynthetic pathways should be bioinformatically explored to support this statement (biosynthesis of Gln from Glu + aminohexoses?).

We thank the reviewer for this interesting question. Glutamine synthetase is indeed present in T. congolense based on transcriptome analysis, and transcripts associated with this gene are present at slightly higher levels (log$_2$ fold change of 1.0) in ex vivo conditions, compared to T. brucei (S1 table). Conversely, abundance of glutamine synthetase transcripts is lower in cultured T. congolense, compared to T. brucei (log2 fold change of -0.2; S1 Table, Fig 8).

Given the lack of divergence from T. brucei in terms of glutamine-derived carbon labelling (Fig 6I), we chose not to focus our work on this amino acid in depth. However, we agree that glutamine synthesis may be worth exploring in the future, especially given that glutamine synthetase is known to play an important role in ammonia detoxification in the related trypanosomatid T. cruzi (PMID: 29320490), a parasite that possesses a higher capacity for amino acid and mitochondrial metabolism than BSF T. brucei.

We note that glutamine is an essential amino acid for T. brucei (PMID: 31028136, 23571546), where it functions as an amino donor. Based on these studies, as well as previous studies that involved culturing of T. congolense (PMID: 7831098, 1852490), we consider glutamine to be essential in this organism. Whilst outside the scope of this manuscript, we agree with the reviewer that glutamine metabolism in general is worth exploring further in BSF T. congolense.

We have added a sentence regarding glutamine synthetase in the relevant portion of the revised manuscript (lines 525-528): "Transcriptomics analysis showed high expression levels of glutamine synthetase in T. congolense, compared to T. brucei, under ex vivo conditions only, suggesting ATP-dependent generation of glutamine may occur in the parasite under these conditions (S2 Table)."

Line 532, Proline is essential for both, PCFs and BSFs in T. brucei. BSFs do not metabolize it to glutamate in an appreciable way. But it is required for protein synthesis and it is not biosynthesized by T. brucei.

This has been amended, the sentence now reads as follows (lines 530-531): "Proline is an essential carbon source for PCF T. brucei and is required for protein synthesis in BSF T. brucei, although it is not a required supplement in BSF medium."

Our rationale for this sentence is that the requirement of proline in BSF T. congolense medium contrasts that of BSF T. brucei, which may be of interest to further study.

Line 539, this amino acid did not contribute in an appreciable way...

This has been amended with a slight variation. We felt the following was applicable: "…labelling showed that this amino acid did not contribute meaningfully to the biosynthesis of other metabolites."

Line 550, It is better to say that is the majoritary role of Asn, since it is not possible to know if below the current detection thresholds other metabolites can come out in the future and some other functions can be found.

We agree with the point the reviewer makes, and apologise for overstating our results. We have amended the sentence and it now reads as follows: "This indicates that, as with proline, asparagine uptake is required principally for protein synthesis in T. congolense."

Line 597, What does it means exactly that acetate/acetyl metabolism is highly active at the level of gene expression?

We apologise to the reviewer for our lack of clarity. In this sentence, we meant to state that at the level of transcriptome, genes associated with this area of metabolism have a high transcript abundance in T. congolense compared to T. brucei. We apologise that this wasn't clear, and have amended the sentence to state the above. The sentence now reads as follows (lines 605-606):

“Several genes associated with acetate/acetyl-CoA metabolism were highly expressed in T. congolense compared to T. brucei (Fig 7C).”

Line 672, This first paragraph is mostly repeated from Introduction, and could be deleted.

This manuscript contains a lot of detail, and we believe it would be helpful to the reader to recap some aspects at this stage of the paper. Whilst we have not deleted the introductory paragraph entirely, we have shortened it and hope this satisfies the reviewer’s request.

Reviewer #3:

There are a few grammatical and numerous punctuation things that need to be rectified.

We apologise for the grammatical and punctuation errors in our submission, we have attempted to rectify this in our resubmission.

Do not feel as though Fig 8 is required, could be supply fig, as the data is presented in Table 1.

We have moved Fig 8 to supplementary (S9 Fig).

In table 1 legend LCFA is define as an abbreviation, but not used in the table?

This has been amended, we have removed the LCFA abbreviation, apologies for this error. In addition, we have added abbreviations for acronyms that are used in table 1.

Line 712/713 22:4 to 22:4, should be 22:5

This has been amended.
